# Muscle wasting assessment tools for prostate cancer

**DOI:** 10.1038/s41598-022-08501-9

**Published:** 2022-03-18

**Authors:** Alan Espinosa-Marrón, Aquiles Rubio-Blancas, Christian Aníbal Quiñones-Capistran, Anais Camacho-Zamora, Itzel Salcedo-Grajales, Ana Paula Bravo-García, Maria T. Bourlon, Ricardo A. Castillejos-Molina, Julie-Alexia Dias, María del Pilar Milke-García

**Affiliations:** 1 Department of Epidemiology, Harvard T.H. Chan School of Public Health, Boston, MA USA; 2 Universidad Autónoma de la Ciudad de México, Mexico City, Mexico; 3 Department of Nuclear Medicine, National Cancer Institute, Mexico City, Mexico; 4 National Institute of Public Health of Mexico, Cuernavaca, Morelos Mexico; 5 Division of Nutrition, National Institute of Medical Sciences and Nutrition “Salvador Zubirán”, Mexico City, Mexico; 6 Department of Hematology and Oncology, National Institute of Medical Sciences and Nutrition “Salvador Zubirán”, Mexico City, Mexico; 7 Department of Urology, National Institute of Medical Sciences and Nutrition “Salvador Zubirán”, Mexico City, Mexico; 8 Department of Biostatistics, Harvard T.H. Chan School of Public Health, Boston, MA USA

**Keywords:** Urological cancer, Disease prevention, Body mass index, Physical examination, Geriatrics

## Abstract

Prostate cancer and its treatment may induce muscle wasting. Body composition and muscle functionality are rarely assessed in patients with prostate cancer from developing countries due to the limited availability of high-quality equipment for routine diagnosis. This cross-sectional study evaluated the association between several simplistic techniques for assessing muscle mass and function with a more complex standard of reference for muscle wasting among Mexican men with prostate cancer. Muscle wasting was highly prevalent, yet it was presumably associated with aging rather than cancer and its treatment itself. The restricted availability of specific equipment in clinical settings with technological limitations supports using unsophisticated techniques as surrogate measurements for muscle wasting. The left-arm handgrip dynamometry displayed the highest correlation with the standard of reference and exhibited an acceptable predicted probability for muscle estimation. Combining several simplistic techniques may be preferable. We also developed and internally validated a manageable model that helps to identify elderly patients with prostate cancer at risk of muscle depletion and impairment. These findings promote the early recognition and treatment of muscle wasting alterations occurring among older adults with prostate cancer.

## Introduction

In men, prostate cancer (PC) is the second most frequently diagnosed neoplasm and the fifth cause of death worldwide^1^. According to the Global Burden of Disease, PC is also the leading cause of cancer mortality among Mexican men^2^. Yet, limited guidance exists for low- and middle-income countries for PC comorbidities screening^1^.

PC and its treatment may induce muscle wasting, a condition associated with nutritional and metabolic alterations, physical impairment, poor quality of life, reduced tolerance to treatments, and shorter survival^3^. Given these detrimental clinical repercussions, it is imperative to promote the early recognition and treatment of muscle wasting alterations occurring during PC. Several methods to assess muscle loss or muscle function had been used in subjects with PC: dual-energy X-ray absorptiometry (DEXA), isometric exercises as a reference for dynamic muscle strength and endurance, and gait speed test^4^. Similarly, evidence suggests that handgrip dynamometry provides precise measurement of strength and muscular endurance among men treated for PC^5^.

Bioelectrical impedance analysis (BIA) has also been used for body composition assessment, and specifically in cancer^6^. This non-invasive method delivers a low-frequency electrical current based on the principle that fluid and cellular structures provide different electrical resistance as it passes through the system. Additionally, vector analysis of impedance—commonly referred to as bioelectrical impedance vector analysis (BIVA)—enables further examination of body composition. BIVA relies on a graphical representation where impedance (Z) is plotted as a vector from its components R (X-axis) and Xc (Y-axis) after being standardized by height. This BIVA RXc z-score graph is divided into four quadrants to classify body composition within the 75% and 95% tolerance ellipses. These quadrants represent (*i*) high cell mass at the top left, (*ii*) low cell mass (referred to as cachexia by some authors) located at the bottom right; (*iii*) edema, bottom left, and (*iv*) dehydrated, top right^6^. This approach also provides a bioelectrical marker referred to as phase angle, where a value between 5 and 7° indicates high cell membrane integrity^7^, reflecting a high cellularity.

Despite its relevance for identifying muscle wasting, body composition and muscle functionality are rarely assessed in patients with PC in developing countries. This is mainly attributed to the limited availability of high-quality equipment that facilitates routine diagnosis (e.g., DEXA, computerized tomography, and magnetic resonance imaging), together with the unawareness of the importance of its assessment. Therefore, this study analyzed multiple techniques requiring minimum equipment and training as proxy measurements for muscle wasting or impaired functionality. We further compare these simplified techniques with Bioelectrical Impedance Vector Analysis (BIVA) as a surrogate estimation for muscle wasting.

## Results

This study included 278 individuals with PC. Missing data were randomly identified in 4.7% of the observations and therefore imputed using the overall sample mean method^8^.

**Figure 1:**
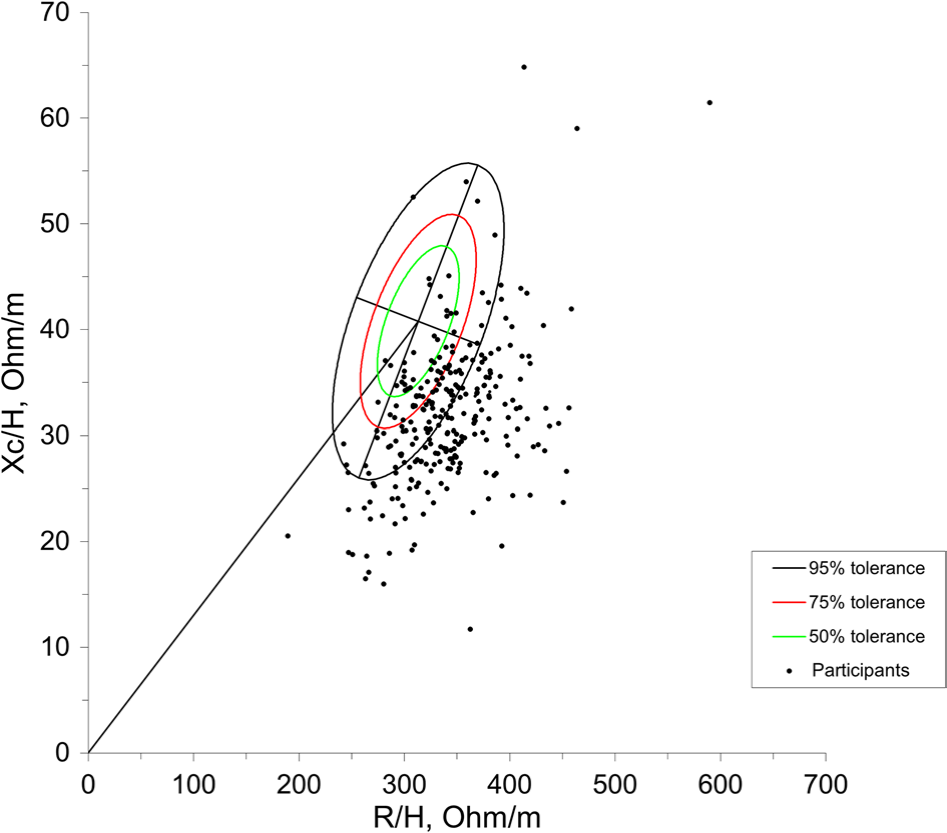
BIVA RXc z-score graph for participants with prostate cancer. Quadrants represent (*i*) top left: high cell mass, (*ii*) bottom right: low cell muscle mass; (*iii*) bottom left: edema, (*iv*) top right: dehydration.

**Figure 2:**
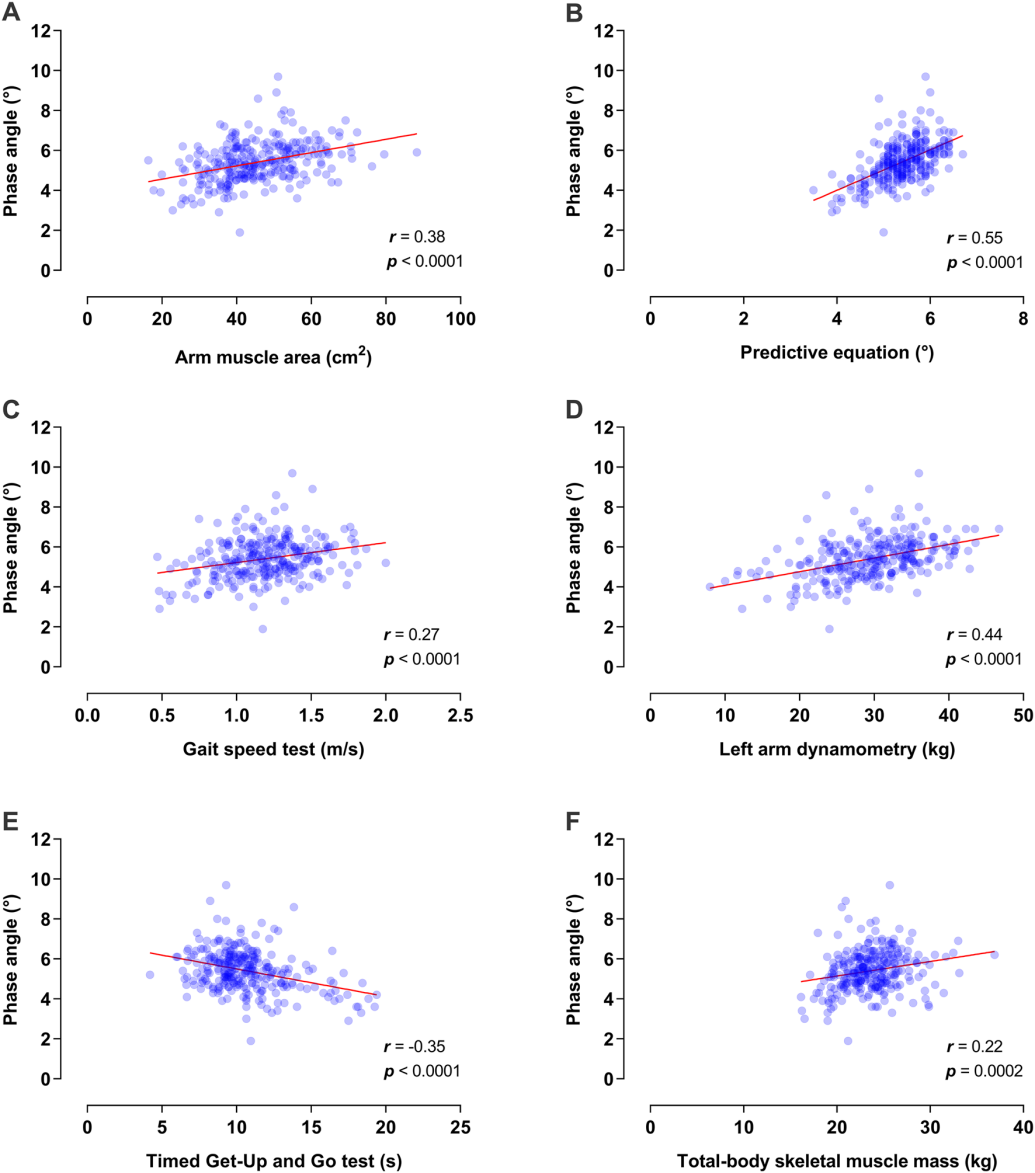
Relationship between alternative tests and BIVA for muscle wasting assessment among subjects with prostate cancer. **(A)** Scatter plot and Pearson correlation between arm muscle area (cm^2^) and phase angle. **(B)** Scatter plot and Pearson correlation between the predicted value through the equation for phase angle (°) and observed phase angle (°). **(C)** Scatter plot and Pearson correlation between Gait speed test (m/s) performance and phase angle. **(D)** Scatter plot and Pearson correlation between Handgrip left arm dynamometry (kg) and phase angle. **(E)** Scatter plot and Pearson correlation between Timed Get-Up and Go test performance (s) and phase angle. **(F)** Scatter plot and Pearson correlation between Total-body skeletal muscle mass (kg) and phase angle.

The analysis revealed that 81% of the participants presented muscle wasting according to BIVA diagnosis (Fig. 1). Aram muscle area, triceps skinfold thickness, time score for both Get-up-and-Go and gait speed performance, and arm dynamometry in patients presenting muscle wasting and in those with adequate muscle mass are depicted in Table 1. Consistently, analyses suggested significant associations between these tests and BIVA (except for triceps skinfold thickness), where the left-arm handgrip dynamometry displayed the strongest correlation (Fig. 2).

**Table 1:** Comparative analysis between subjects' characteristics according to BIVA diagnosis.

	Muscle wasting (n = 226)	Without muscle wasting (n = 52)	p-value^1^
***Biophysical characteristics***			
Age (years)	72.4 ± 7.4	69.1 ± 5.8	**0.0007**
BMI (kg/m^2^)	27.0 (5.1)	27.5 (4.8)	0.6752
Phase angle (°)	5.2 (1.2)	6.6 (0.9)	** < 0.0001**
R/H (Ω/m)^┼^	340.3 ± 41.7	343.4 ± 57.2	0.3258
Xc/H (Ω/m)^┼^	30.5 (6.39)	39.3 (7.01)	** < 0.0001**
Fat mass (%)	34.0 (5.8)	32.2 (6.1)	0.1662
Fat free mass (%)	65.6 (6.7)	67.6 (6.1)	0.4117
Lean dry mass (%)	17.3 (2.2)	17.6 (1.6)	0.2808
***Anthropometric measurements***			
Arm circumference (≤ 5 percentile)	3.2%	0.7%	0.9347
Arm muscle area (≤ 5 percentile)	30.3%	5.1%	0.1291
Calf circumference (≤ 5 percentile)	1.4%	0%	0.3271
Triceps skinfold thickness (≤ 5 percentile)	0%	0.4%	**0.0394**
Total-body skeletal muscle mass (kg)	23.6 (3.4)	23.5 (5.7)	0.8428
Skeletal muscle mass index (kg/m^2^)	8.6 (1.3)	8.5 (1.9)	0.7550
***Biochemical markers***			
Serum albumin (g/dl)	4 (1)	4 (1)	0.12
***Muscle functionality***			
Timed Get-Up-and-Go test (s)	10.4 (2.8)	9.9 (2.0)	**0.0376**
Gait speed tests (m/s)	1.2 ± 0.3	1.3 ± 0.2	**0.0218**
Left arm dynamometry (kg)	29.5 (9.0)	32.7 (7.7)	**0.0026**
Right arm dynamometry (kg)	31.6 ± 7.0	33.8 ± 6.3	**0.0359**
***Pre-existing disease***			
Obesity (according to BMI)	19.4%	3.2%	0.2722
Hypertension	54.7%	45.3%	0.2182
Diabetes	25.8%	24.5%	0.8511
Metastatic prostate cancer	20.0%	15.1%	0.4134
***Treatment for prostate cancer***			
Hormone replacement	41.3%	36.5%	0.5254
Radiotherapy	23.6%	27.5%	0.5580
Chemotherapy	8.9%	13.2%	0.3395
Radical prostatectomy	54.2%	62.3%	0.2889

*BIVA* bioelectrical impedance vector analysis, *BMI* body mass index.

Significant values are in bold.

^1^Obtained by Student's *t*-test or Mann–Whitney *U*, according to distribution Data presented as means ± SD or medians (IQR). For proportion comparisons, a ${\chi }^{2}$ test was computed.

^┼^These strong associations are expected as part of the definition of wasting used to classify participants.

Table 2 describes the logistic models on the association between muscle wasting according to BIVA and alternative tests. Statistically significant associations only persisted for arm dynamometry after adjusting for age. Results revealed that for every additional kilogram of handgrip pressure, the odds of having an adequate muscle mass increased 7%. Among those subjects whose arm dynamometry resulted in less than 30 kg, the crude odds of presenting muscle wasting according to BIVA were 2.11 times that of adults with a proficiency equal to or greater than 30 kg (95% CI 1.16–3.85; *p* = 0.0149). In this analytic approach, the association was also diminished when controlling for age (${\mathrm{OR}}_{\mathrm{MH}}$= 1.87, 95% CI 1.01–3.46; *p* = 0.0458) and stratified odds ratios resulted in 1.91 (95% CI 0.95–3.83; *p* = 0.0684) and 1.75 (95% CI 0.47–6.48; *p* = 0.4037) for older (≥ 65 years old) and younger individuals (< 65 years old), respectively. We did not identify alterations in the magnitude association induced by other confounders (i.e., smoking status, level of physical activity).

**Table 2:** Logistic regression models on the association between adequate muscle mass according to alternative tests and BIVA.

Alternative testing options	Crude model			Adjusted model^1^		
	β-coefficient	OR (95% CI)	p-value	β-coefficient	OR (95% CI)	p-value
Predictive model (°) ^2^	–	–	–	1.271	3.57 (1.92–6.96)	** < 0.0001**
Arm dynamometry (kg)	0.081	1.08 (1.03–1.14)	**0.001**	0.066	1.07 (1.00–1.12)	**0.039**
Arm muscle area (cm^2^)	0.028	1.03 (1.01–1.06)	**0.032**	0.036	1.04 (0.99–1.08)	0.087
Arm muscle area (> 5 percentile)	0.514	1.67 (0.87–3.3)	0.132	0.373	1.33 (− 0.29 to 1.08)	0.284
BMI (kg/m^2^)	− 0.016	0.99 (0.91–1.06)	0.693	− 0.001	0.99 (0.83–1.18)	0.908
Calf circumference (cm)	0.013	1.01 (0.92–1.12)	0.791	− 0.038	0.96 (0.80–1.17)	0.698
Gait speed test (m/s)	1.293	3.64 (1.21–11.38)	**0.021**	0.457	1.58 (0.36–6.96)	0.544
Timed Get-Up-and-Go test (s)	− 0.160	0.85 (0.74–0.97)	**0.022**	− 0.585	0.94 (0.79–1.12)	0.504
Total-body skeletal muscle mass (kg)	0.016	1.02 (0.92–1.12)	0.739	− 0.060	0.94 (0.81–1.08)	0.405
Triceps skinfold (cm)	− 0.053	0.95 (0.88–1.02)	0.133	− 0.062	0.94 (0.85–1.03)	0.189

Significant values are in bold.

^1^Adjusted for age as a potential confounder on the prostate cancer—muscle wasting association.

^2^The equation includes age as a predictive variable.

Notably, there were no associations between muscle impairment and treatment alternatives [OR for hormone replacement = 1.00 (95% CI 0.45, 2.20), *p* = 0.99; OR for radiotherapy = 0.73 (95% CI 0.24, 1.48), *p* = 0.35; OR for chemotherapy = 0.63 (95% CI 0.24, 1.65), *p* = 0.35; OR for radical prostatectomy = 0.61 (95% CI 0.29, 1.29), *p* = 0.20], time (in years) since diagnosis [OR 1.06 (95% CI 0.99, 1.14) *p* = 0.11], time (in years) of treatment exposure [OR 1.00 (95% CI 0.73, 1.38) *p* = 0.99], and physical activity level [OR for moderate physical activity = 0.90 (95% CI 0.47, 1.72), *p* = 0.74; OR for vigorous physical activity = 0.53 (95% CI 0.21, 1.30), *p* = 0.16). Considering the low/inactive category as the reference]. The mean muscle mass did not vary according to PC therapy.

**Figure 3:**
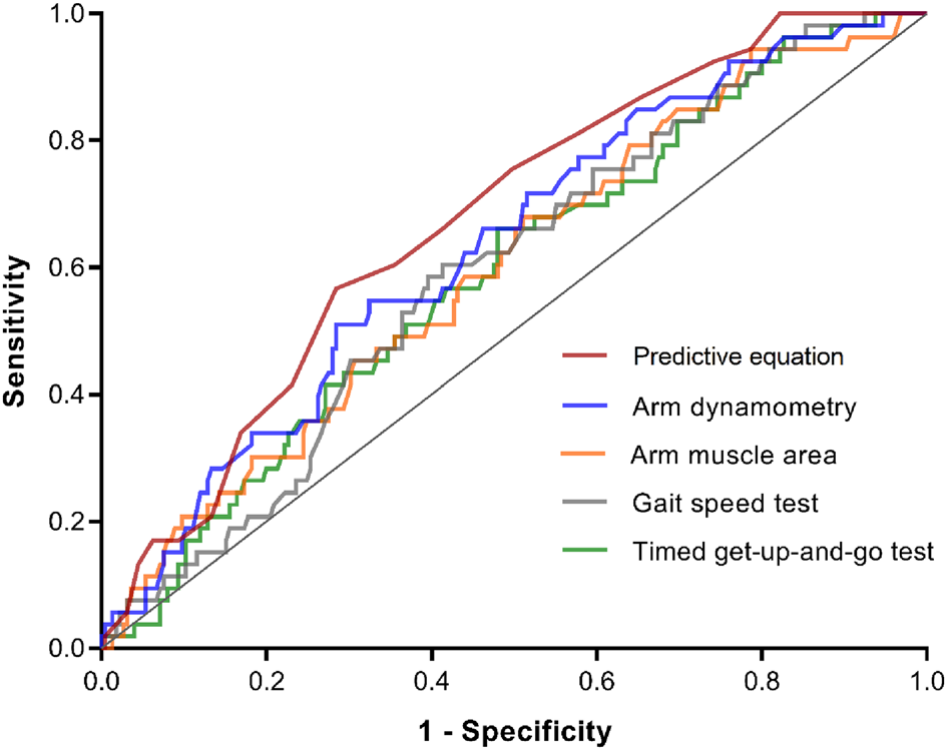
ROC curves for tests' diagnostic performance based on BIVA “cachexia” classification. Phase angle predictive model (°): AUC = 0.68 (0.60–0.75; *p* < 0.0001). Arm dynamometry (kg): AUC = 0.63 (0.55–0.71; *p* = 0.003). Arm muscle area (cm^2^): AUC = 0.60 (0.52–0.68; *p* = 0.025). Gait speed test (m/s): AUC = 0.59 (0.52–0.67; *p* = 0.033). Timed Get-Up and Go test (s): AUC = 0.59 (0.51–0.67; *p* = 0.038).

Figure 3 presents the AUC of alternative tests that displayed a significant performance. The combination of various simplistic diagnostic techniques enhances precision upon the estimator. Our polytomous prediction model for the estimation of phase angle based on simplistic techniques (Eq. 1) exhibited a significant linear association (*p* < 0.0001), accounted for 71% overall accuracy with a standard error of the mean of 0.14° between the observed and predicted phase angles (PA) [calibration: 5.4° (1.3°) *vs.* 5.5° (0.7°), respectively; *p* = 0.394], and displayed an adjusted-*R*^2^ value of 0.31. The 0.68 C-index indicated moderate capacity for discrimination. The measures included in the predictive model (Eq. 1) as continuous variables after cross-validation were age, Timed Get-Up-and-Go test (TUG), mid-upper arm circumference (MUAC), triceps skinfold thickness (TSFT), and left-hand dynamometry (LHD).

1 $$\mathrm{Phase\, angle}= 4.434\,-\,\left(0.024 \,\times\, \mathrm{Age}\right)\,-\,\left(0.050\, \times\, \mathrm{TUG}\right)\,+\,\left(0.094\, \times\, \mathrm{MUAC}\right)\,-\,\left(0.048 \,\times\, \mathrm{TSFT}\right)\,+\,(0.034 \,\times\, LHD)$$

## Discussion

Body composition is seldom assessed in patients with PC in developing countries such as Mexico. This is mainly attributed to the limited availability of high-quality equipment that facilitates routine diagnosis (e.g., DEXA, computerized tomography, and magnetic resonance imaging), together with the unawareness of the importance of its assessment. We identified that muscle wasting prevalence was as high as 81% in a sample of Mexican adults with PC. It is therefore necessary to include muscle wasting screening as a complementary physical examination to prevent clinical outcomes that may increase mortality rates among patients with PC^9^. Muscle wasting has been consistently associated with a decrement in long-term survival, along with perioperative and oncologic complications in patients receiving radical prostatectomy, radiotherapy, or systemic therapy for PC^10^. The inclusion of muscle wasting assessment procedures as complementary screening in patients undergoing PC is fundamental for preventing morbidity and mortality risks^11^.

Therefore, this study explored different diagnostic techniques requiring minimal equipment and training as alternative proxy measurements for muscle loss. Our results suggest that left handgrip dynamometry (kg), arm muscle area (cm^2^), gait speed test (m/s), and Timed Get-Up-and-Go test (s) may be acceptable and timely predictors for evaluating PC patients. However, after controlling for age as a confounder, the association between the diagnostic tests and muscle wasting was strongly attenuated. Thus, in this study muscle loss could be presumably attributed to aging and not to cancer cachexia. This outcome may also explain why both the severity of cancer and PC treatment did not seem to be associated with muscle function impairment. Further research may be needed to assess the contribution of each factor on the body composition alterations in PC patients.

Our study found that the left handgrip dynamometry displayed the highest correlation with BIVA's phase angle. Previous studies have also shown that PC decreases handgrip strength, nevertheless this impairment being attributed to hormone replacement^9,12^. Hormone replacement—also known as androgen deprivation therapy (ADT) is an important treatment for patients with prostate cancer. However, ADT can cause significantly skeletal muscle loss that may increase the risk of non-cancer mortality^13^. Forty-one percent of participants were concomitantly receiving ADT when enrolled in our study (41.3% presenting muscle wasting and 36.5% without muscle wasting). Our subgroup analysis according to treatment did not replicate prior results, as muscle mass from participants receiving hormone deprivation was comparable to those exposed to other treatments, and regression models did not corroborate an association between ADT (or any other treatments) and muscle depletion. On the other hand, evidence is consistent with our finding of lower handgrip strength associated to aging^14^.

BMI is a valuable measure to assess all-cause mortality risk in older adults^15^. However, one concern regarding BMI as a predictor of overall mortality is the inability to discriminate among type and distribution of body tissues. Our results suggest that this parameter was not an accurate proxy measurement for muscle wasting according to BIVA. This is not unexpected, as the strength of correlation coefficients between BMI and body components is typically attenuated with increasing age^16^, which could be particularly emphasized in this study evaluating elderly volunteers. Other methods for estimating body composition also report several limitations restraining their use under specific circumstances (i.e., elevated cost, complex equipment, trained personnel). Therefore, stating that BMI is an inferior parameter for our purpose seems to be unjustified; instead, the selection of the most adequate method to assess body composition will depend on the purpose of this evaluation and should be therefore tailored accordingly to the population's characteristics.

Our most important contribution was having developed and validated a prediction equation that estimates phase angle that may be applied in a real-world framework. Literature indicates that phase angle is linearly related to muscle mass and strength reduction, thus representing a marker for muscle loss in elderly patients. It also is related to muscle body composition, its integrity, and functionality—providing evidence of patients' nutritional and clinical condition^17,18^. This novel formula is a simple and inexpensive alternative that contributes to the multidimensional evaluation of older adults at risk of muscle wasting in preventive care and geriatric medicine. Literature further recommends a PA threshold of 5.2 as a criterion to identify nutritional risk based on muscle impairment in cancer patients^18^.

We acknowledge some methodological limitations associated with the cross-sectional nature of our design and relatively small sample size, possibly reducing analytic precision. Future studies assessing muscle wasting among older Mexican adults with PC will also benefit from cancer severity biomarkers. Still, we hypothesize no other associations as our findings suggest that muscle impairment is related to aging rather than cancer cachexia, yet interactions may also be present.

Phase angle is inversely related to muscle mass and strength in the elderly and could therefore be considered a fair indicator of muscle wasting^17^. Our predictive equation simplifies the estimation of this parameter but does not provide the RXc z-score graph, commonly used for clinical interpretation and patients' classification. Furthermore, every predictive model's positive and negative predictive values depend on the prevalence of a specific disease in a certain population. A high prevalence of muscle wasting—such as the one we reported—considerably increases the positive predictive value of our equation but may yield a different parameter in other populations with lower muscle impairment prevalence. We carefully conducted a *k*-fold cross-validation process to enhance its extension so that the model was independently developed and validated without overfitting. This analytic approach also provides a model performance comparable to what could be observed in a real‐world framework^19^. However, our model would further benefit from external validation by using an independent database.

Evaluating participants' nutritional status is as important as measuring other clinical, anthropometric, and biochemical indicators. Even when we presented serum albumin as a surrogate biomarker of the nutritional status (Table 1), our study focused exclusively on comparing techniques to facilitate muscle mass wasting identification. Cross-sectional studies–such as the present study–are prone to confounding and reverse causation when evaluating diet, as these do not capture within-person variation throughout time. Prospective analysis reliant on validated dietary tools along with repeated measures could complement our results by accounting for effect measure modification introduced by diet quality. In practice, we encourage professional, evidence-based nutritional counseling as a fundamental procedure of PC treatment. The Summary of the Third Expert Report from the World Cancer Research Fund offers dietary and nutritional information for PC management^20^.

In the same sense, longitudinal evaluation of skeletal muscle is of critical relevance during prostate neoplasm. Our results could benefit from prospective studies using our formula with periodic evaluations to account for muscle changes occurring during PC and its treatment.

Derived from the unavailability of a DEXA, computerized tomography (CT) or magnetic resonance imaging equipment (MRA), this study's most remarkable limitation was the use of an imperfect standard of reference for muscle mass estimation. Literature suggests that bioimpedance measures are not accurate predictors of biological outcomes: their potential usefulness relies on the inclusion of weight, height, sex, and age as covariates in prediction models that are commonly incorporated in the analysis. However, impedance per se could add error towards the estimation^21^.

To address the previous limitation, we conducted secondary analyses considering handgrip dynamometry as the surrogate referent measurement. According to preceding evidence^14^, it seems reasonable to use this strength measure as a biological response for muscle wasting. The associations between each diagnostic technique and handgrip dynamometry are presented in Fig. S1 and Table S1 included in the Supplementary findings section. These results are consistent with our initial findings. Hence, our message remains constant: unsophisticated techniques are reliable alternatives to estimate muscle impairment in older adults with PC—especially in the absence of a more accepted measure for lean mass (DEXA, CT, MRA).

We recognize that a large body of evidence has correlated DEXA and alternative measures to assess muscle integrity and functionality^22^. Nevertheless, we failed to identify studies estimating muscle wasting using different techniques and generating a predictive model among Mexican patients undergoing PC. Given that Mexico is a multicultural, diverse, and vast country, studies performed in different countries might not entirely suit the Mexican context. It is, therefore, essential to generate country-specific data to promote the early recognition and treatment of muscle wasting when a more accepted measure for lean mass is not available. Our study also encourages culturally sensitive research and reveals challenges and opportunities while doing so.

In conclusion, muscle wasting is common among Mexican men with PC, yet it is presumably associated with aging rather than cancer and its treatment itself. Its assessment through unsophisticated techniques and mostly by handgrip dynamometry approximates its diagnosis through BIVA. The limited availability of specific equipment or devices in the clinical setting supports its use as the preferred diagnostic alternative; combining several simple techniques may be preferable. Here, we propose a manageable algorithm that predicts phase angle as a bioelectrical marker to identify elderly Mexican patients with PC at risk of muscle depletion and impairment.

## Methods

A cross-sectional study was conducted among Mexican men with PC to evaluate the association between different techniques for assessing muscle mass and function with the BIVA as a proxy for muscle wasting.

The minimum required sample size estimation was based on the parameters and size effect of muscle mass proportion identified in our pilot study^23^. We considered a Cohen's *d* of 0.45 with an *α* error probability of 0.05, a power of 0.95, and an allocation ratio of 1. The calculated sample size resulted in 272 participants recruited through printed and electronic advertisements.

Eligibility criteria included men with PC of any histological variant receiving chemotherapy, radiotherapy, hormone replacement therapy, or radical prostatectomy who attended the Urology outpatient service at the National Institute of Medical Sciences and Nutrition "Salvador Zubirán" (Mexico) between 2018 and 2020. We excluded candidates with dementia, terminal disease, neurological or motor deficits, using mobility-aid accessories, cardiovascular disease, high-performance athletes, and those patients presenting edema, dehydration, or any other condition affecting body composition. Participants provided written informed consent. Demographic and health information (including biomarkers) were drawn from the medical records. Physical activity and sedentary behavior were evaluated using the International Physical Activity Questionnaire (IPAQ), classifying levels of physical activity as low/inactive, moderate, and vigorous^24^.

According to national and international regulations, the protocol adhered to the STROBE guidelines for observational studies and was approved by the Ethics Committee from the National Institute of Medical Sciences and Nutrition "Salvador Zubirán" (approval number uro-844-13/13-1) in agreement with the Mexican Secretary of Health and the Declaration of Helsinki. All methods were thereby performed under this approved protocol describing all relevant guidelines and adhered to national and international regulations for human research.

### Bioelectrical impedance analysis

Tetrapolar electrical bioimpedance was used for body composition assessment (Quantum X, RJL SYSTEMS). Participants were evaluated in standardized conditions as indicated by the manufacturer^25^. Bioelectrical Impedance Vector Analysis (BIVA) approach proposed by Piccoli was then used as the reference proxy measurement for lean body mass^26^. Impedance (Z) was divided into height-adjusted (H) resistance (R) and reactance (Xc). Individual phase angle points calculated as (Xc/R) × 180°/π) were plotted on an R/H-Xc/H graph based on bivariate tolerance ellipses validated for the Mexican population^27^. A conventional impedance analysis complemented the assessment to obtain fat-free mass, fat mass, and body water based on previously validated regression models^28^. Moreover, total-body skeletal muscle mass was estimated using the equation proposed by Janssen^29^. Skeletal muscle mass index was also calculated by dividing the resulting skeletal muscle mass (kg) by the square of the height (m^2^)^30^.

### Anthropometric parameters

Weight and height were assessed with a mechanical column scale (SECA 700) and used for body mass index (BMI) determination. Arm, waist, hip, and calf circumferences were measured with a Lufkin metal tape, and the triceps skinfold thickness was measured with a Lange caliper. All anthropometric procedures adhered to the International Society for the Advancement of Kinanthropometry (ISAK) methodology^31^. Mid-arm muscle mass (mm^2^) was calculated based on arm circumference and triceps skinfold measurements, as proposed by Frisancho^32^.

### Muscle functionality assessment

Handgrip strength was measured with a T-18 Mark Smedley III dynamometer. Participants were asked to adopt the standard testing position approved by the American Society of Hand Therapists^33^. According to the European Working Group guidelines on Sarcopenia in older people, a handgrip strength < 30 kg is the cutoff value for muscle wasting^34^.

We evaluated functional mobility through the Timed Get-Up-and-Go test. The testing area was set up by measuring 3 m from a straight-backed armchair's front legs with a seat height of $\sim$ 46 cm. Participants were instructed to sit with their backs against the chair and arms comfortably placed on the armrests. At the word 'go,' patients stood and walked the specified distance at an average pace, turned around, returned to the chair, and sat down. The stopwatch was started on the word 'go' and stopped when the participant returned to the starting position^35^.

A 4-m gait speed test was conducted separately to complement the analysis. A flat, unobstructed course was established to perform this assessment, and a line mark was set at 4 m. Participants were instructed to walk to the end of the course at their usual speed. Timing with a stopwatch started when the participant began to move and stopped when the participant's first foot completely crossed the 4-m line^36^.

### Statistical analysis

Subjects were classified as presenting or not muscle wasting according to BIVA diagnosis. Shapiro–Wilk tests were performed to assess normality, and groups' characteristics were further compared through Student's *t* or Mann–Whitney *U* tests, as appropriate. Regression analyses were conducted to evaluate the influence of both preexisting diseases and PC treatment therapies on muscle wasting, and the prevalence of these conditions was contrasted between groups using a chi-square test.

We used logistic regression models to estimate the association between muscle wasting according to BIVA and alternative testing options (arm dynamometry, arm muscle area, calf circumference, gait speed test, timed get-up and go test, skeletal muscle mass index, and total-body skeletal muscle mass), controlling for age as a confounder. Pearson's correlation coefficients complemented the analysis. Each technique result was then classified as a binary outcome (muscle wasting or adequate muscle mass) and stratified according to the level of the confounder (younger or older adults). Ratio estimates were obtained for each specific stratum, and Mantel‐Haenszel estimators were computed as the population referents. Tests' diagnostic performance was assessed based on the receiver operating characteristic (ROC) curve and the area under the curve (AUC) as its summary index.

A predictive equation was generated using a linear regression model with *k*-fold cross-validation to estimate BIVA's phase angle based on the alternative testing options. This procedure consisted of randomly dividing the original cohort into a training dataset containing 80% of the participants, assigned at random to 10 independent sub-samples for models' development. The remaining 20% of the sample served as testing data for cross-validation. The rationale of dividing the entire dataset into random training and testing sets is that the predictive model can be independently developed and cross-validated without overfitting, so that the model's performance would be comparable to that observed in a real‐world framework^19^. The final equation performance was further assessed based on bias, precision, and accuracy. Bias represented the standard error of the mean between predicted and observed values, precision expressed the goodness of fit (*R*^2^), and the accuracy reflected the proportion of people with predicted phase angles within ± 15% of the observed value^37^. We also used the PROBAST (prediction model risk of bias assessment tool) for assessing the risk of bias and applicability of the resulting equation^38^. Predictive performance was determined according to discrimination (the extent to which predicted outcome discriminate between participants with and without the outcome, represented by the C-index) and calibration (the extent to which the predicted outcome correspond to the observed outcome) as recommended in the TRIPOD (transparent reporting of a multivariable prediction model for individual prognosis or diagnosis)^39^.

Statistical analyses were conducted using RStudio and GraphPad Prism version 9.

## Supplementary Information

Supplementary Information.

## Data Availability

The dataset analyzed in the present study is available from corresponding author on reasonable request.
