# Supplementary material for: Muscle wasting assessment tools for prostate cancer
Source: Sci Rep. 2022 Mar 18;12:4662. doi: 10.1038/s41598-022-08501-9 (PMC8933481; doi:10.1038/s41598-022-08501-9)
Supplement: Supplementary file 1 — Supplementary Information. [file 41598_2022_8501_MOESM1_ESM.docx]

**Supplementary findings**

**Muscle wasting assessment tools for prostate cancer**

Alan Espinosa-Marrón^1^, Aquiles Rubio-Blancas^2^, Christian Aníbal Quiñones-Capistran^3^, Anais Camacho-Zamora^4^, Itzel Salcedo-Grajales^5^, Ana Paula Bravo-García^5^, Maria T Bourlon^6^, Ricardo A. Castillejos-Molina^7^, Julie-Alexia Dias^8^, María del Pilar Milke-García*^5^.

| **Table S1.** Logistic regression models on the association between adequate muscle mass according to alternative tests and left handgrip dynamometry. | | | | | | | | | | | | |  |
| --- | --- | --- | --- | --- | --- | --- | --- | --- | --- | --- | --- | --- | --- |
|  |  | **Crude model** | | | | |  | **Adjusted model**^1^ | | | | |  |
| *Alternative testing options* |  | β*-coefficient* |  | *OR (95% CI)* |  | p*-value* |  | β*-coefficient* |  | *OR (95% CI)* |  | p*-value* | |
| Arm muscle area (cm^2^) |  | 0.038 |  | 1.04 (1.02 – 1.06) |  | **0.001** |  | 0.022 |  | 1.02 (1.00 – 1.05) |  | 0.068 | |
| BMI (kg/m^2^) |  | 0.122 |  | 1.01 (0.95 – 1.08) |  | 0.390 |  | -0.027 |  | 0.97 (0.91 – 1.04) |  | 0.422 | |
| Calf circumference (cm) |  | 0.130 |  | 1.14 (1.05 – 1.23) |  | 0.289 |  | 0.059 |  | 1.06 (0.97 – 1.16) |  | 0.198 | |
| Gait speed test (m/s) |  | 2.736 |  | 15.43 (5.58– 42.68) |  | **< 0.001** |  | 2.045 |  | 7.73 (2.62 – 22.76) |  | **< 0.001** | |
| Timed Get-Up-and-Go test (s) |  | -0.306 |  | 0.74 (0.66 – 0.83) |  | **< 0.001** |  | -0.205 |  | 0.81 (0.72 – 0.92) |  | **0.001** | |
| Total-body skeletal muscle mass (kg) |  | 0.249 |  | 1.28 (1.17 – 1.41) |  | **< 0.001** |  | 0.178 |  | 1.19 (1.08 – 1.32) |  | **< 0.001** | |
| Triceps skinfold thickness (cm) |  | 0.013 |  | 1.01 (0.96 – 1.07) |  | 0.480 |  | -0.257 |  | 0.98 (0.92 – 1.03) |  | 0.381 | |
| ^1^Adjusted for age as a potential confounder on the prostate cancer – muscle wasting association.  ^2^ The equation contemplates age as a predictor variable. | | | | | | | | | | | | |  |

**
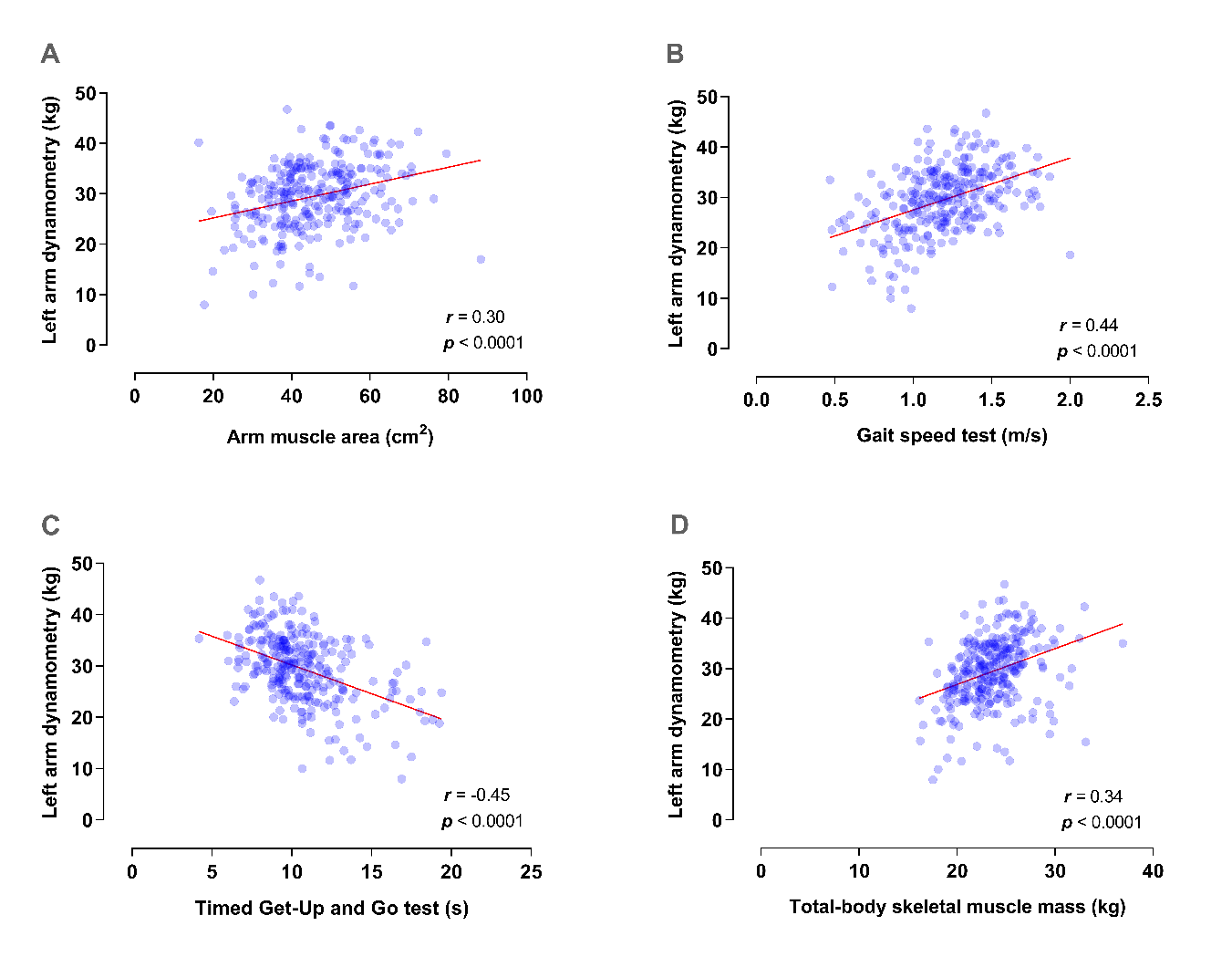
**

**Figure S1.** Relationship between alternative tests and left handgrip dynamometry for muscle wasting assessment among subjects with prostate cancer. **(A)** Scatter plot and Pearson correlation between arm muscle area (cm^2^) and left handgrip dynamometry. **(B)** Scatter plot and Pearson correlation between Gait speed test (m/s) performance and left handgrip dynamometry. **(C)**  Scatter plot and Pearson correlation between Timed Get-Up and Go test performance (s) and left handgrip dynamometry. **(D)** Scatter plot and Pearson correlation between Total-body skeletal muscle mass (kg) and left handgrip dynamometry.
